# Immunotherapy in Hepatocellular Carcinoma with Portal Vein Tumour Thrombosis: From Poor Prognosis to Curative-Intent Strategies

**DOI:** 10.3390/cancers18040627

**Published:** 2026-02-14

**Authors:** Luca Marzi, Rodolfo Sacco, Luisa Siciliani, Saveria Lory Crocè, Mauro Giuffrè, Cristina Stasi, Chiara Turri, Monica Zoeschg, Andrea Mega

**Affiliations:** 1 Department of Gastroenterology, Bolzano Regional Hospital (Südtiroler Sanitätsbetrieb—Azienda Sanitaria dell’Alto Adige SABES-ASDAA), 39100 Bolzano-Bozen, Italyandrea.mega@sabes.it (A.M.); 2 Gastroenterology and Digestive Endoscopy Unit, Foggia University Hospital, 71122 Foggia, Italy; 3 Division of Clinical Immunology and Infectious Diseases, Fondazione IRCCS Policlinico San Matteo, 27100 Pavia, Italy; 4 Department of Medical, Surgical, and Health Sciences, University of Trieste, 34127 Trieste, Italy; 5 Clinica Patologie Fegato, Azienda Sanitaria Universitaria Giuliano Isontina (ASUGI), 34125 Trieste, Italy; 6 Department of Life Science, Health and Health Professions, Link Campus University, 00165 Rome, Italy; c.stasi@unilink.it

**Keywords:** hepatocellular carcinoma, portal vein tumour thrombosis, immunotherapy, local therapies

## Abstract

Background/Objectives. Globally, hepatocellular carcinoma (HCC) is the sixth most prevalent cancer and represents the third leading cause of cancer-related mortality. Despite advances in diagnosis and treatment, the overall prognosis of HCC remains poor, largely due to late-stage presentation and high rates of vascular invasion. Portal vein thrombosis constitutes a significant complication of HCC, with an occurrence rate of 35–50%. Portal vein tumour thrombosis (PVTT) represents an aggressive form that is closely linked to decreased patient survival. The incidence of PVTT increases over time, from approximately 21% in the first year after diagnosis to nearly 46% by the third year, highlighting its high prevalence and progressive nature. The introduction of immunotherapy has marked a paradigm shift in the management of HCC. Immune checkpoint inhibitors, particularly those targeting the PD-1/PD-L1 and CTLA-4 pathways, have demonstrated durable responses and significant survival benefits in certain patients with advanced HCC. Furthermore, emerging evidence suggests that combining immunotherapy with local or systemic therapies, such as trans arterial chemoembolization, hepatic arterial infusion chemotherapy, radiotherapy or tyrosine kinase inhibitors, may further enhance antitumor efficacy by modulating the tumour microenvironment and promoting synergistic immune activation. Method: This narrative review provides a comprehensive evaluation of immunotherapy in patients with HCC and PVTT, with a focus on its efficacy as both monotherapy and in combination with other treatment modalities in terms of tumour response, progression-free survival, and overall survival. It also addresses safety, patient selection, and emerging strategies to optimize outcomes in this high-risk population. Conclusions: The combination of multiple therapies could improve the patient’s prognosis by achieving objective response rate of almost 82%, disease control rate of 97% and progression free survival of 12.9 months with quadruple therapy (HAIC-TACE combined with targeted and immunotherapy).

## Simple Summary

Hepatocellular carcinoma (HCC) is the sixth most common cancer and the third leading cause of cancer-related mortality. One of the complications of HCC is malignant portal vein thrombosis. Portal vein tumour thrombosis (PVTT) is an aggressive form of cancer closely linked to reduced patient survival and with few treatment options. The introduction of immunotherapy has marked a paradigm shift in the management of HCC and it is precisely in this patient setting that it could play an important role in downstaging. This narrative review provides a comprehensive assessment of immunotherapy in patients with PVTT-HCC.

## 1. Introduction

Hepatocellular carcinoma (HCC) ranks as the sixth most prevalent cancer worldwide and represents the third leading cause of cancer-related mortality [1]. Portal vein thrombosis occurs in up to 35–50% of advanced HCC cases and it can be malignant or non-tumoral [2]. The incidence of portal vein tumoral thrombosis (PVTT) in HCC ranges from 21% at one year to 46% at three years [2] and is associated with a poor prognosis [2]. Other factors that may influence prognosis are the degree or extent of PVTT due to impaired liver function and limited therapeutic options. Currently, there is no global consensus or established protocols regarding the optimal management of HCC with associated PVTT. The Barcelona Clinic for Liver Cancer (BCLC) classifies HCC patients with PVTT as stage C, indicating an advanced stage, and limiting treatment recommendations for these patients to systemic therapy [3]. This represents a significant limitation, as a more refined stratification would allow the identification of patients with limited PVTT extension who may be suitable candidates for more aggressive and individualized treatment strategies. In recent years, there has been an increase in the availability of therapeutic options for PVTT-HCC patients [4]. Types of treatment include systemic therapy, trans-arterial chemoembolization, surgical resection, stereotactic body radiotherapy, trans-arterial radioembolization, and liver transplantation [4,5]. An ideal therapy for each patient necessitates a multidisciplinary approach [5]. This article presents a narrative review on the treatment of PVTT, focusing on immunotherapy.

## 2. Materials and Methods

Although this is a narrative review and not a systematic one, we adopted a structured search strategy to ensure transparency. A literature review was conducted using the following PubMed medical subject heading (MeSH) terms: “portal vein tumour thrombosis”, “portal vein tumour thrombosis”, “hepatocellular carcinoma”, “liver tumour”, “immunotherapy”, “immune checkpoint inhibitors”. We prioritized peer-reviewed clinical studies, systematic reviews, meta-analyses, and major guidelines. Eligible studies included peer-reviewed original research articles and review studies involving patients with HCC that specifically addressed PVTT. Conference abstracts, editorials, commentaries, animal or in vitro studies, non-English publications, and studies not specifically addressing PVTT in HCC were excluded. All duplicated records were removed. Study selection was conducted in two stages: initial title and abstract screening to exclude irrelevant studies, followed by full-text screening to assess eligibility based on the predefined criteria (Figure S1). Data were extracted using a standardized form, collecting information on authorship, year of publication, country and study setting, study design, sample size, type of portal vein thrombosis, diagnostic methods, treatment strategies, and clinical outcomes, and the results were summarized descriptively.

## 3. Classification and Prognostic Model for the Treatment of Portal Vein Tumour Thrombosis

The PVTT East classifications, Japanese VP classification and Cheng’s classification in China, can help to select and stratify the PVTT treatment [6,7]. Japanese VP classification distinguished four grades of PVTT and has been widely used. Chinese Cheng’s classification is preferred to combine the imaging indistinguishable early-stage tumour thrombus (type I) and divide the imaging distinguishable main portal vein tumour thrombus (type III) from superior mesenteric tumour thrombus (type IV), which is more conducive to the guide the surgical and comprehensive treatment strategies. However, the recommended treatment options for patients with PVTT in the West (Europe and America) and the East (Asia–Pacific region) are very different [8,9,10,11,12,13]. In parallel with the existing PVTT classification systems, an Eastern-developed prognostic model has been introduced to optimize the selection of patients eligible for hepatic resection [14]. The East Hepatobiliary Hospital portal vein tumour thrombosis (EHBH-PVTT) scoring system is commonly used in China for the selection and prognostic stratification of patients with HCC and PVTT who are candidates for hepatic resection [8,14]. This system, based on total bilirubin, alpha-fetoprotein, tumour diameter, and the presence of satellite lesions, has been validated in large multicenter cohorts and allows the identification of patients with limited PVTT (first-order branches or main portal trunk) who may benefit from margin-negative surgical resection [14].

Immune checkpoint inhibitors (ICIs) have significantly changed the treatment of HCC, particularly in advanced cases. The combination of atezolizumab and bevacizumab is now a key first-line systemic therapy for unresectable HCC [15]. This regimen uses complementary mechanisms, offering a more targeted and effective approach than traditional tyrosine kinase inhibitors such as sorafenib [15]. Atezolizumab, a monoclonal antibody against PD-L1, blocks the PD-L1/PD-1 pathway, which tumours use to evade immune detection. Blocking this pathway reactivates cytotoxic T lymphocytes, allowing the immune system to target malignant hepatocytes. However, the tumour microenvironment often limits this response due to poor vascularisation and the presence of immunosuppressive cytokines and cells, which hinder T-cell infiltration and function.

Bevacizumab (anti-vascular endothelial growth factor (VEGF) antibody) plays a synergistic role. VEGF not only promotes angiogenesis but also facilitates immunosuppression within the tumour environment by recruiting regulatory T cells and myeloid-derived suppressor cells, and by impairing dendritic cell maturation [16]. By inhibiting VEGF, bevacizumab not only halts tumour neovascularization but also remodels the immune environment, enabling greater T-cell access to the tumour and enhancing the effects of atezolizumab [16]. These theoretical underpinnings were validated in the IMbrave150 trial, a phase 3 randomized controlled study that compared atezolizumab plus bevacizumab (atezo-bev) versus sorafenib in patients with unresectable HCC [16]. Importantly, the trial included a subset of patients with macrovascular invasion, particularly those with segmental or lobar PVTT, though individuals with main trunk PVTT were largely excluded due to the high risk of hepatic decompensation and impaired drug delivery through compromised portal flow [17].

Alternative immunotherapeutic approaches have also demonstrated clinical utility in HCC, notably the combination of Nivolumab and Ipilimumab, which has been investigated in the CheckMate 040 trial [18]. Nivolumab, an anti-PD-1 antibody, shares mechanistic similarities with atezolizumab but targets the PD-1 receptor rather than its ligand. When paired with Ipilimumab (anti-CTLA-4 antibody) this regimen exerts a dual checkpoint blockade—enhancing both T-cell activation in lymphoid organs and effector T-cell function within the tumour. CheckMate 040 included a broader population of patients with advanced HCC, some of whom had prior exposure to sorafenib and macrovascular invasion, including PVT. However, dual immune checkpoint blockade is associated with a higher incidence of immune-related adverse events, necessitating careful patient selection [18]. Thus, it is typically reserved for immune-competent patients with good performance status and the capacity to withstand immune-mediated toxicities.

A third noteworthy approach is the durvalumab plus tremelimumab combination, evaluated in the HIMALAYA trial and now approved as another first-line regimen for unresectable HCC. This unique “STRIDE” regimen (Single Tremelimumab Regular Interval Durvalumab) consists of a one-time priming dose of tremelimumab, a CTLA-4 inhibitor, followed by durvalumab monotherapy, which continuously inhibits PD-L1. This strategy aims to boost the initial T-cell activation while minimizing long-term toxicity associated with sustained CTLA-4 inhibition [19]. The generalizability of these findings is limited by the small sample size of patients with clinically significant PVT. For individuals in whom bevacizumab is contraindicated, including those with a history of variceal bleeding or coagulopathy, the STRIDE regimen serves as a valid, evidence-based alternative [19]. Immune checkpoint inhibitors have fundamentally transformed the management of HCC, with combinations such as atezolizumab plus bevacizumab, nivolumab with or without ipilimumab, and durvalumab plus tremelimumab demonstrating significant survival benefits. In patients with PVTT, particularly those with segmental or lobar involvement, these therapies offer a real opportunity for disease control, and in some cases, downstaging to enable local-regional treatments or surgical intervention. However, the presence of main trunk PVTT, decompensated liver disease, or poor performance status continues to present clinical challenges.

### 3.1. Immune Mechanisms in Hepatocellular Carcinoma and Portal Vein Tumour Thrombosis

Hypothesized mechanisms in the development of PVTT are numerous. While substantial evidence supports that PVTT typically arises from the primary HCC lesion, some studies have suggested that PVTT may, in certain cases, originate from distinct clonal populations [20]. The molecular pathways involved in PVTT formation and progression are highly complex, involving a network of factors such as hepatitis B virus infection, hypoxic tumour microenvironments, cancer stem cells (CSCs), extracellular matrix remodelling, circulating tumour cells, and dysregulated non-coding RNAs [20,21,22,23,24,25].

The immunological mechanisms involved in PVTT in HCC are complex and multifactorial. The immune microenvironment of PVTT is characterized by a predominance of tumour-associated macrophages (TAM), particularly those expressing the C5aR receptor, which promote an immunosuppressive environment [23]. These C5aR+ TAMs inhibit the cytotoxic activity of CD8+ lymphocytes by suppressing Granzyme B production and promote tumour thrombus progression by modulating the local immune response [23]. Another key mechanism is the recruitment of immunosuppressive cells, such as regulatory T cells (Tregs), mediated by signals such as TGF-β and chemokines (e.g., CCL22), which facilitate the immune escape of tumour cells and colonization of the portal system [26]. Furthermore, the presence of erythroid-like transdifferentiated myeloid cells (EDMCs), induced by tumour macrophages via the CCR2 signal, contributes to the impairment of the vascular endothelium, the activation of coagulation (increase in factors such as FX, FVII, fibrinogen) and the promotion of tumour cell migration, aggravating the formation of PVTT [27]. Local hypoxia, typical of the tumour microenvironment, induces the expression of HIF-1α, which promotes epithelial–mesenchymal transition and the production of immunosuppressive cytokines, favouring the expansion of macrophages and Tregs, with further suppression of the anti-tumour immune response [28]. In summary, neoplastic portal thrombosis in HCC is supported by an immune microenvironment dominated by immunosuppressive macrophages, Treg cells, EDMCs, and pro-inflammatory and pro-coagulant signals, which facilitate tumour growth and thrombus formation [23,26,27,28]. Tumour cells exist within a complex, heterogeneous tumour microenvironment (TME), where immune cells play a central and dynamic role [28]. In HCC, this landscape is uniquely shaped by the liver’s dual functions in immunity and tolerance. While extracellular matrix dysregulation and altered focal adhesion have been linked to the development of PVTT, the immune composition of PVTT and its functional relevance remain poorly defined. Recent advances in single-cell technologies—such as scRNA-seq and CyTOF—enable high-resolution analysis of tumour ecosystems and have already revealed key immune and tumour cell heterogeneity in primary HCC [28]. Extending these methods to PVTT may uncover mechanisms driving its formation and progression [28].

### 3.2. Immunotherapy in Main-Trunk PVTT

The management of HCC complicated by main-trunk PVTT, particularly VP4 classification, represents one of the most challenging oncologic scenarios. Among these, the combination of atezo-bev has emerged as the preferred first-line systemic therapy for patients with preserved hepatic function and VP3–VP4 PVTT. This recommendation is now enshrined in leading clinical guidelines, including the 2021 Chinese HCC guidelines, supported by Level Ib, Grade A evidence [28]. This endorsement is primarily rooted in data from the pivotal IMbrave150 trial, a landmark phase III study that fundamentally altered the standard of care for advanced HCC. In this high-risk cohort, 48 patients received atezo-bev, while 25 patients were treated with sorafenib. Although the sample size was limited, the findings were clinically relevant. Median OS reached 7.6 months in the PVTT group treated with Vp4, compared to 5.5 months in the sorafenib group [24]. The hazard ratio (HR) for OS was 0.62 (95% CI: 0.34–1.11), which did not achieve statistical significance (descriptive *p* = 0.10) but suggests a possible therapeutic advantage. In the Vp4 population, progression-free survival (PFS) increased from 2.8 months in patients treated with sorafenib compared to 5.4 months of patients treated with atezo–bev, aligning with trends observed in the broader trial population [24]. Importantly, this outcome signalled not only the efficacy of immune checkpoint blockade in vascular invasion contexts but also validated the tolerability of anti-VEGF therapy in patients with high thrombotic burden—provided that pre-treatment screening and prophylactic measures against variceal bleeding are employed [24]. This evidence is confirmed by several case reports and a clinical study [21,23,29,30,31,32,33]. Similar case reports have been described with other combinations (PD-1 inhibitor and VEGFR inhibitor) [34] or other regimens [35]. Supporting these observations, a study of 22 consecutive patients with Vp3/4 PVTT treated with atezolizumab plus bevacizumab reported a median survival of 15.0 months and 1- and 2-year survival rates of 62.7% and 49.3%, respectively, with markedly superior objective response rates (91.7% vs. 10.0%) and 2-year survival (78.6% vs. 20.0%) in patients with preserved liver function compared with those with impaired function, demonstrating that this regimen is particularly effective in advanced HCC with PVTT when liver function is well preserved [33]. Park et al. [25] conducted a retrospective comparison of atezo-bev versus lenvatinib as first-line therapy in HCC patients with PVTT, finding no significant differences in OS, PFS, or disease control rate [25]. Another study, including 110 patients with unresectable HCC treated with PD-1 inhibitors, including 34 patients with macrovascular invasion of the portal vein or inferior vena cava, was compared with 34 patients without vascular tumour thrombi [36]. The importance of vascular response as an independent positive predictor of OS has been documented in a study of 34 patients (13 treated with PD-1 monotherapy and 21 treated with combination therapy with Tyrosine Kinase Inhibitors—TKIs) with macrovascular invasion of the portal vein or inferior vena cava [36].

### 3.3. Combination Immunotherapy and Locoregional Therapy

The integration of locoregional and systemic treatments has emerged as a particularly dynamic area of progress. Although systemic therapy (immunotherapy and TKIs) may have revolutionized the prognosis of patients with HCC and TTVP, combining it with locoregional therapy could have a greater impact in terms of response or survival.

Some clinical cases illustrate how combination conversion therapy can reduce the stage of extensive tumour thrombi [37]. However, real-world data caution that the addition of transarterial chemoembolization (TACE) does not uniformly benefit patients with main portal vein invasion, underscoring the need for careful patient selection [38]. Regimens combining TACE with TKIs or PD-1 inhibitors consistently outperform TACE alone, improving response rates, OS, and progression-free survival (PFS) [39] without significantly increasing severe adverse events [40]. Comparative studies suggest that Hepatic arterial infusion chemotherapy (HAIC)-based combinations (especially HAIC with lenvatinib (LEN) and PD-1 blockade) may offer even greater efficacy than TACE-based regimens, including in patients with complex vascular anatomy such as arterioportal fistulas [41]. Likewise, TACE–HAIC combined with LEN or camrelizumab prolongs survival compared with TACE alone, with only modest increases in manageable toxicity [42]. Emerging biomarker research suggests that inflammatory cytokines such as IL-6, IL-17, VEGF, and IFN-α may help predict treatment response, potentially guiding future patient selection [35]. In a cohort of 164 patients, HAIC-LEN-PD-1 therapy doubled median OS and significantly extended PFS relative to LEN-PD-1 alone, with substantially higher ORR and DCR despite slightly increased but manageable toxicity [43]. Another multicentre propensity-adjusted analysis showed that adding TACE to TKI + ICI therapy significantly improved ORR, PFS, and OS, and remained an independent predictor of survival, without increasing high-grade adverse events [44]. Similar benefits have been observed with transarterial radioembolization (TARE) combined with atezo-bev, which achieves meaningful tumour reduction while maintaining a favourable safety profile [45].

The combination of immunotherapy with locoregional therapies could also have an impact in terms of neoadjuvant therapies, to the point of providing curative interventions (resection or liver transplant). Among combination strategies, triple therapy (TACE plus a TKI and a PD-1 inhibitor) has gained traction as a neoadjuvant option for resectable PVTT-HCC. This approach reduces microvascular invasion, decreases satellite nodules, and lowers AFP levels, translating into superior survival compared with surgery alone [46]. Multicentre data report high response rates, prolonged PFS, and median OS approaching two years after resection, reinforcing the central role of multimodal therapy in this population [47]. Yu et al. further demonstrated that triple therapy markedly improves OS, PFS, ORR, and PVTT response compared with dual systemic therapy, without increasing grade toxicity [48]. A meta-analysis by Du et al. corroborated these findings, confirming superior survival, response rates, disease control, and downstaging with triple therapy, again without added severe adverse events [49]. A 2025 meta-analysis by Xuan et al. highlighted that neoadjuvant strategies—including radiotherapy, transcatheter arterial chemoembolization, hepatic artery infusion chemotherapy, and concurrent chemoradiotherapy—improved survival outcomes compared with upfront surgery, without increasing perioperative complications [50]. Parallel advances in surgical technique have further expanded the therapeutic window. Approaches such as longitudinal incision with transverse suturing and angle-to-straight conversion have shown excellent long-term portal vein patency and effective thrombectomy, particularly among patients achieving pathological complete response, highlighting the feasibility of salvage surgery following combination therapy [51].

Innovative multimodal protocols continue to push therapeutic boundaries. The “SITS” regimen (sorafenib combined with ICIs, TACE, and Stereotactic body radiation therapy—SBRT) has demonstrated substantial tumour shrinkage and survival benefits, creating opportunities for subsequent surgery or transplantation [52]. Sequential strategies, such as HAIC followed by TACE and targeted and immune therapies, have achieved exceptionally high objective response and disease control rates with manageable toxicity, offering a promising option for patients with otherwise refractory disease [53]. Other studies are described in Table 1, sorted by level of evidence.

### 3.4. Combination Immunotherapy and Radiotherapy

Radiotherapy (RT) appears to enhance the immunogenic effects of ICIs in HCC with PVTT. Kim et al. reported a near-complete response in a patient with advanced HCC and massive PVTT treated with RT (50 Gy in 10 fractions) plus atezo-bev [68]. In a propensity-matched cohort of 72 patients, the addition of RT to TKI and PD-1 therapy significantly improved clinical outcomes. Median OS increased from 8.2 to 15.6 months, and PFS improved from 5.2 to 8.1 months, with toxicity remaining within acceptable limits [69]. The most pronounced benefits were observed in patients with main-trunk PVTT and Child–Pugh A liver function. Furthermore, RT combined with TKI and PD-1 therapy was an independent predictor of both OS and PFS [69]. Similarly, a multicentre study involving 113 patients demonstrated that PVTT-directed RT followed by ICIs plus bevacizumab significantly prolonged PFS (9.6 vs. 5.4 months), improved ORR (48.9% vs. 27.7%), and increased DCR (97.9% vs. 82.98%) compared to ICIs plus bevacizumab alone, without additional toxicity [70]. Prospective data from a 30-patient multicentre trial demonstrated that intensity-modulated RT combined with atezo–bev achieved an ORR of 76.6%, with median OS and PFS of 9.8 and 8.0 months, respectively, highlighting strong synergistic effects in extrahepatic PVTT [71].

A retrospective chart review identified 62 patients with HCC and PVTT (Vp3 in 47% and Vp4 in 45%) treated with RT (61% stereotactic body RT or 39% fractionated RT) and concurrent immunotherapy. CTP A patients had significantly improved outcomes, while no survival differences were seen between single- and multiagent immunotherapy. Median progression-free survival was 3.7 months, and overall survival was 7.7 months. Retrospective cohort of 71 HCC patients with PVTT comparing RT + HAIC + TKI + ICI versus HAIC + TKI + ICI. The combination including RT improved PFS and OS without increasing adverse events, suggesting it is an effective and safe non-surgical option for HCC with PVTT [72].

Another retrospective analysis was conducted on patients with Vp4 PVTT who underwent hypofractionated radiotherapy (5 Gy × 5) and immunotherapy with atezo-bev or durva-treme. The results were compared between Vp4 patients treated with RT, Vp4 patients without RT, and patients without Vp4 invasion. Of the eight Vp4 patients who underwent hypofractionated RT with immunotherapy, high ORR were observed with OS comparable to that of patients without Vp4 invasion and superior to that of Vp4 patients who did not receive RT [73]. These promising results must be interpreted cautiously given small sample sizes and potential selection bias favouring patients with better performance status and liver function for RT. Prospective randomized data are needed.

### 3.5. Clinical Trials

Several prospective clinical trials are currently exploring intensified multimodal strategies for HCC with PVTT. The PATENCY II trial (NCT06669377) is investigating a comprehensive strategy that integrates TACE, immune checkpoint inhibitors, targeted therapy, and irradiated stent placement to maintain portal vein patency in patients with Vp4 (main trunk) PVTT [74]. This large multicentre study plans to enrol 444 patients and will evaluate safety, efficacy, survival outcomes, and long-term stent patency. The NCT05166239 trial [75] is a phase II study comparing HAIC combined with lenvatinib and PD-1 inhibitors versus lenvatinib and PD-1 therapy alone in advanced hepatocellular carcinoma (HCC) with PVTT, with 6-month progression-free survival (PFS) as the primary endpoint. The BRAVE Study (NCT07062055) is assessing a triplet regimen of QL1706 (anti-PD-1/CTLA-4), bevacizumab, and stereotactic body radiotherapy (SBRT) in patients with BCLC-C HCC with PVTT or limited metastases [76]. This multicentre phase II trial aims to recruit 46 patients and will assess PFS, OS, and radiologic response. Collectively, these trials demonstrate increasing global interest in combining immunotherapy, targeted agents, and radiotherapy or interventional techniques to improve outcomes in this complex patient population (Table 2).

## 4. Discussion

We reviewed studies demonstrating the effect of combining ICIs, TKIs, and locoregional therapies in patients with HCC with PVTT. This represents a significant shift in treatment approaches. This represents a significant shift in therapeutic approaches. Furthermore, case reports [35] indicate that integrating immunotherapy with TKIs or anti-PD-1 agents can produce complete or durable responses in selected patients. Retrospective analyses [36] further support these findings by identifying vascular response as an independent prognostic factor for improved overall survival. Overall, this evidence underscores the importance of treating both intrahepatic disease and macrovascular invasion. While systemic monotherapy remains foundational, a growing body of evidence underscores the potentiated efficacy of triple approaches, integrating immunotherapy, TKIs, and locoregional therapies such as HAIC, RT, or TACE. These combinations reflect a broader shift toward biological convergence, exploiting immunomodulatory and vascular-disruptive mechanisms in tandem to overcome the immunosuppressive microenvironment of PVTT. Although in different populations, triple therapy (TACE + TKI + PD-1) appears to be superior to dual therapy or TACE alone [44,46,47,48,49,69]. HAIC-based combinations [41,43,60,62,65] show longer OS and PFS rates than TACE-based combinations, with ORR > 50%. Quadruple regimens [53] show an ORR of almost 82%, Disease Control Rate of 97% and PFS of 12.9 months. Neoadjuvant therapy [46,47,49] improves OS and eRFS and allows for surgery with better outcomes. These findings indicate that combining immunologic, antiangiogenic, and locoregional therapies leads to better tumour control and improved intrahepatic and vascular responses, while maintaining an acceptable safety profile. About RT added to TKI + PD-1, there is a significant improvement in OS and PFS compared to systemic therapy alone, with a more marked effect in PVTT of the main trunk and in Child–Pugh A [69].

RT directed at PVTT followed by ICIs + bevacizumab increases PFS, ORR, and DCR without increasing toxicity [34]. Intensity-modulated RT + atezo–bev shows high ORR (76.6%) with PFS of 8 months and OS of 9.8 months, indicating strong synergy, especially in extrahepatic PVTT [71].

Importantly, PVTT shows much higher radiosensitivity than the primary tumour, with pathological complete response rates of 78.6% in treated thrombi versus 21.4% in primary lesions [77]. Overall, these results suggest that RT enhances systemic antitumor immunity and renders the PVTT microenvironment particularly responsive to therapy.

### 4.1. Clinical Implications and Treatment Algorithm

**Figure 1:**
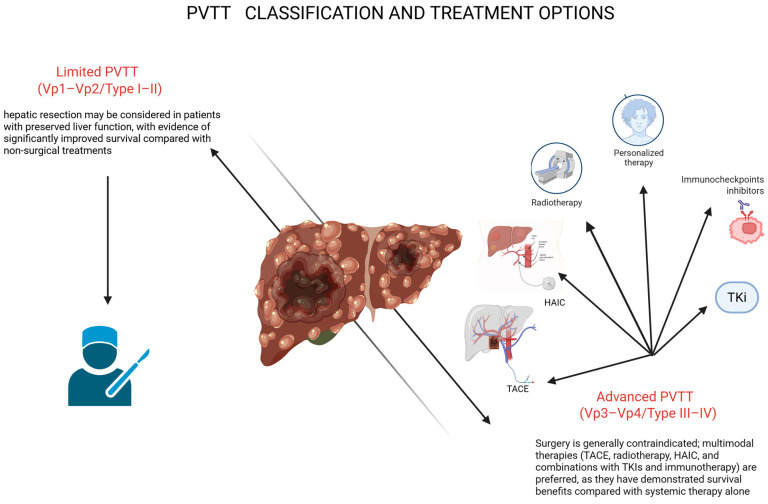
Classification of portal vein tumour thrombosis (PVTT) and corresponding treatment strategies. Limited PVTT (Vp1–Vp2/Type I–II) may allow consideration of hepatic resection in patients with preserved liver function, whereas advanced PVTT (Vp3–Vp4/Type III–IV) is generally managed with multimodal therapies, including TACE, radiotherapy, HAIC, and combinations with TKIs or immunotherapy, which offer superior survival compared with systemic therapy alone. Treatment selection is guided by PVTT extent, liver function, and overall tumour burden. TACE: Transarterial Chemoembolization; HAIC: Hepatic Arterial Infusion Chemotherapy; TKI: Tyrosine kinase inhibitor. Created in BioRender. Siciliani, L. (2025) https://BioRender.com/1g6zly8.

Based on current evidence, treatment of HCC with PVTT should be individualized according to the extent of portal vein involvement and liver function (Figure 1).

**Figure 2:**
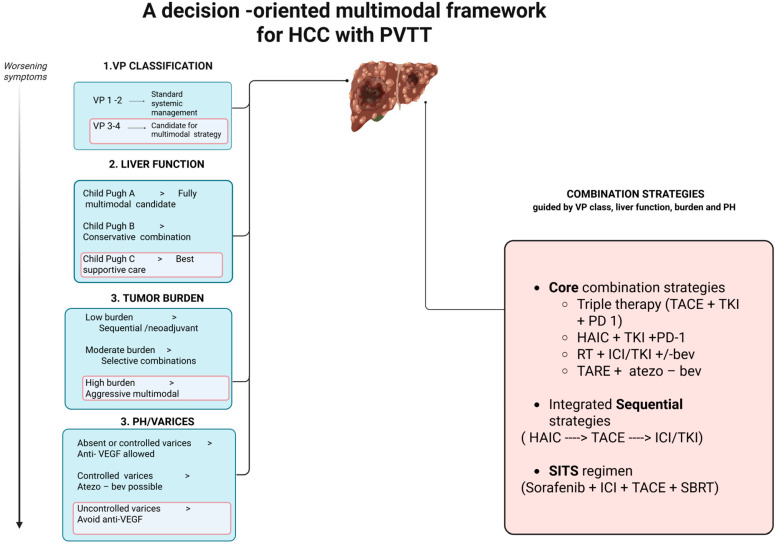
A decision-oriented multimodal framework based on VP Classification, Liver Function, Tumour Burden and Portal Hypertension. Created in BioRender. TACE: Transarterial Chemoembolization; HAIC: Hepatic Arterial Infusion Chemotherapy; TKI: Tyrosine kinase inhibitor; anti-VEGF: anti-vascular endothelial growth factor; TARE: Transarterial Radioembolization; PD-1: Programmed Cell Death Protein 1; ICI: Immune Checkpoint Inhibitor; SBRT: Stereotactic Body Radiation Therapy. Created in BioRender. Siciliani, L. (2025) https://BioRender.com/1g6zly8.

In patients with limited PVTT (Vp1–Vp2) and preserved liver function, aggressive multimodal treatment may be considered. Combination strategies incorporating locoregional therapy (HAIC or TACE) with a TKI and an ICI have shown high response rates and may allow tumour downstaging and conversion surgery in selected cases. For patients with advanced PVTT (Vp3–Vp4) and Child–Pugh A liver function, atezo-bev remains the standard first-line systemic therapy. However, HAIC-based combination regimens are emerging as potential alternatives, particularly in patients with dominant intrahepatic disease, although their optimal role requires further validation. In cases of Vp4 PVTT involving the main portal vein trunk, RT targeting the PVTT, followed by systemic therapy, may improve portal flow and treatment tolerance, offering a possible survival benefit in this high-risk population. These recommendations are largely based on retrospective studies from Asian cohorts, and prospective randomized trials are needed to confirm efficacy, define optimal sequencing, and establish standardized treatment algorithms. We propose the following treatment approach (Figure 2).

### 4.2. Limitations of Current Evidence

Several important limitations of the existing literature preclude definitive conclusions regarding the optimal management of HCC with PVTT. First, there is a pronounced geographic bias, with more than 80% of available data derived from East Asian populations, where underlying liver disease etiology, clinical practice patterns, and access to specific treatment modalities (such as HAIC) differ substantially from those in Western countries. As a result, the external validity and generalizability of these findings to non-Asian populations remain uncertain. Second, most published studies are retrospective in nature, which inherently introduces selection bias, confounding, and treatment allocation bias. Patients selected for aggressive multimodal therapies are often younger, have better preserved liver function, and lower tumour burden, which may overestimate treatment efficacy compared with unselected real-world populations. In addition, heterogeneity in institutional expertise and treatment protocols further complicates interpretation of outcomes. Third, there is a lack of standardized response assessment criteria specifically designed for PVTT. Conventional radiologic criteria such as Response Evaluation Criteria in Solid Tumours (RECIST) or mRECIST primarily focus on intrahepatic tumour burden and may not adequately capture changes in vascular tumour thrombus, portal vein recanalization, or hemodynamic improvement. Given the aggressive nature of PVTT and the evolving use of immunotherapy-based combinations, short follow-up may underestimate long-term survival, durability of response, and delayed immune-related or vascular toxicities. Longer observation periods are essential to fully characterize outcomes and to determine whether early benefits translate into sustained survival advantages.

## 5. Future Directions

Despite significant therapeutic advances, several key questions remain unresolved in the management of HCC with PVTT. The optimal sequencing of systemic and locoregional therapies has yet to be clearly defined, particularly as combination and triplet regimens become more widely adopted. In addition, the role of predictive and prognostic biomarkers, including PD-L1 expression, alpha-fetoprotein (AFP) levels, and emerging immune-based signatures, requires further clarification to enable more precise patient selection. For example, there are specific biomarkers that can predict benefits from combination therapies in patients with HCC and PVTT. High tumour mutational burden (TMB), defined >10 mutations per megabase, is associated with improved overall survival and response rates to ICI-based combination therapies in advanced HCC, including those with PVTT [78]. This is supported by meta-analyses showing superior 1-year overall survival for patients with high TMB compared to moderate TMB, and improved objective response rates and disease control rates with ICI combinations versus monotherapy [78]. Immune gene signatures—especially high expression of intratumoural CD8+ T cell density, and CD274 (PD-L1)—are linked to better clinical outcomes with atezo-bev, as demonstrated in molecular analyses of phase III trials [79]. The presence of glucose-6-phosphate dehydrogenase (G6PD) positive and cell surface vimentin positive circulating tumor cells, which reflect epithelial–mesenchymal and ferroptosis activity, is associated with poor response to targeted immunotherapy and increased risk of PVTT formation. This dual biomarker can stratify patients for risk and monitor therapeutic efficacy [80]. These biomarkers are emerging tools for precision management, but prospective validation in large cohorts is still needed. Their use can help identify patients most likely to benefit from combination regimens and guide therapy selection in HCC with PVTT.

Safety and efficacy data are also limited for patients with moderately impaired liver function (Child–Pugh B7–8), a population frequently underrepresented in clinical trials but commonly encountered in real-world practice. Finally, the cost-effectiveness and resource implications of increasingly complex multimodal treatment strategies remain largely unexplored and will be critical for informing clinical decision-making and policy development.

## 6. Conclusions

Recent evidence suggests that combination therapies are generally superior to monotherapy or dual therapy in HCC complicated by PVTT. Approaches integrating locoregional treatments (HAIC or TACE) with systemic therapy, including ICIs and TKIs or anti-VEGF agents, demonstrate promising outcomes. These strategies not only improve response rates but may also enable downstaging of disease and subsequent salvage surgery in selected patients, thereby offering potential curative benefit. Hepatic arterial infusion chemotherapy combined with ICIs, with or without TKIs, has demonstrated high response rates even in cases involving main trunk PVTT. However, most supporting data are derived from retrospective cohorts, primarily within East Asian populations. The safety profiles of combination regimens remain manageable, and the addition of ICIs does not substantially increase the incidence of severe adverse events in most reports. This underscores the importance of careful patient selection based on liver function and performance status. Despite these promising results, several gaps persist. Prospective randomized trials specifically investigating PVTT in the context of ICI-based combination therapy are limited, with most evidence originating from retrospective or observational studies. The optimal sequencing, timing, and patient selection criteria for interventions such as HAIC, TACE, radiotherapy, or combinations with different ICIs, TKIs, or anti-VEGF agents have yet to be clearly defined. Additionally, biomarkers predictive of response in PVTT, including characteristics of the immune microenvironment and PD-L1 expression, remain underexplored. Further research is needed to assess long-term outcomes, quality of life, and the impact of combination therapies on liver function preservation.

## Figures and Tables

**Table 1:** Studies described according to level of evidence.

Levels of Evidence	Study	Design/Population	Intervention (s)	Outcomes Relevant to PVTT/Macrovascular Tumour Thrombus	Notes & Safety
	Lu D [54]	Systematic review and meta-analysis. 17 trials involving 3070 HCC patients comparing systemic therapy alone versus systemic therapy combined with HAIC BCLC B-C, subgroup analyses including PVTT.	Systemic therapy ± HAIC	Improve OS (HR 0.52), PFS (HR 0.54), ORR, DCR	AEs ≥ 3 grade
**Meta-analysis/RCT/analysis post hoc**	Jiang M [55]	Bayesian network meta-analysis	Triple therapy (combinations of locoregional therapy—HAIC, TACE, or radiotherapy—with targeted therapy (e.g., TKIs) and immunotherapy (PD-1/PD-L1 inhibitors) vs. targeted + immunotherapy	Improve OS e PFS In PVTT subgroups ORR 50–60%, DCR 80–90%	Triple therapy increased grade 3–4 AEs compared with systemic therapy alone (73.5% vs. 39.4%; *p* < 0.001), but toxicities were considered clinically manageable
**Retrospective studies with propensity score matching (PSM)**	Zou X [56]	160 patients	TACE + LEN + PD-1 vs. TACE + LEN	OS 23.5 vs. 18.3 months, PFS 7.5 vs. 4.3 months Higher disease control rate (80.0% vs. 56.7%) and ORR (38.6% vs. 24.5%) in the triple therapy group	Safety: AEs acceptable Multivariate analysis identified Child–Pugh grade, PVTT classification, and inflammatory cytokines (IL-6, IL-17, IFN-α, VEGF) as predictors of survival outcomes.
	Liu B [57]	Single-centre of 197 (BCLC stage B/C)	TACE + HAIC + TKI + ICI vs. HAIC + TKI + ICI	OS 20.8 vs. 14.2 mesi; PFS 15.4 vs. 10.6 mesi; ORR 54%	AEs ≥ G3 28%
	Li Y [58]	Multicenter with large (>10 cm) HCC complicated by major portal vein tumour thrombosis	Triple therapy (HAIC + lenvatinib + PD-1 inhibitor) vs. HAIC	OS (21.2 months vs. 6.6 months), PFS (7.4 months vs. 3.0 months), Intrahepatic tumour ORR: 57.7% vs. 19.7%, PVTT ORR: 62.0% vs. 21.1%, DCR: 91.5% vs. 59.2% (all *p* < 0.001)	Grade 3/4 AEs were similar between groups
	Wu HX [59]	Retrospective cohort (*n* = 160), Child–Pugh A/B, BCLC stage C, PVTT VP2–4 (≈50% VP3)	TACE + LEN + PD-1 vs. TACE + LEN	TACE + LEN + PD-1 associated with significantly better outcomes; inflammatory cytokines (IL-6, IL-17, VEGF, IFN-α) may predict survival	Safety profile manageable; patient selection based on liver function and disease stage
	Liu Q [60]	37 pts with advanced HCC + PVTT in main trunk	mFOLFOX-based HAIC + TKI + ICI	PVTT response: CR 18.9%, PR 56.8%, ORR 75.68%. 6-month survival 89%, 1-year 66%. Median OS 15.8 month.	AEs commonly pain, thrombocytopenia from oxaliplatin. Child–Pugh score significant predictor of OS.
	Fu Y [61]	53 Len-PD1 group and 89 HAIC-Len-PD1 group	FOLFOX-HAIC + Lenvatinib and PD1 inhibitor	OS were 13.8 months in the Len-PD1 group and 26.3 months in the HAIC-Len-PD1 group. PFS 11.5 months versus 5.5 months	
	Cao F [62]	Real-world cohort, HCC + PVTT	HAIC + TKI + PD-1 inhibitor	Vp4 type PVTT treated with PD-1 inhibitor increased OS by 6.0 months (*p* = 0.04).	Incidence of grade 3–4 AEs was similar between groups (30.9% vs. 19.7%, *p* = 0.09). However, two patients in the HTP group experienced immune treatment-related fatalities.
**Retrospective studies without propensity score matching (PSM)**	Kuwano A [63]	48 advanced HCC with PVTT	Atezo + Bev vs. HAIC	No significant difference in OS	The survival of advanced HCC patients with PVTT is intricately linked to the preservation of liver function
	Li SQ [64]	Retrospective study	TACE + Lenvatinib + PD-1 inhibitors	OS, PFS, response rates improved	AEs: fever (31.7%), hypertension (26.8%), fatigue (24.4%), abnormal liver function (63.4%) and decreased appetite (21.9%). No treatment-related mortality occurred.
	Li J [65]	Single-centre retrospective study	HAIC + anti-PD-1	OS 14.9 mo and PFS 6.9 mo	A total of 31.1% of grade 3–4 AEs were reported
	Fu S [66]	Retrospective analysis, Vp3 and Vp4 or tumours occupying >50% of the liver.	HAIC-FOLFOX: 5-fluorouracil, leucovorin, oxaliplatin) combined with lenvatinib and PD-1 inhibitors	PFS 8.8 mo, OS 14.3 mo ORR 52.7% by RECIST 1.1, DCR 95.6%	AE: 94.5%, but none were fatal. Safety was considered well tolerated overall.
	Lee SK [67]	Retrospective, multicenter cohort study of 97 HCC patients with PVTT without metastasis (37 received Ate/Bev, 60 TACE + RT	Atezo-Bev vs. TACE + RT (50 Gy/5 fractions).	One-year survival was significantly higher with Ate/Bev than TACE + RT. One-year PFS and OS were improved with Ate/Bev ORR and DCR were similar between groups	No significant differences in baseline characteristics between groups. Safety/adverse events were assessed per CTCAE v5.0, but specific toxicity results were not detailed in the abstract.

**Table 2:** Clinical trials.

NCT/Identifier	Title/Short Name	PVTT Classification Included	Intervention/Combination	Phase/Design	Sample Size/Status	Key Endpoints
NCT06669377 (PATENCY II) [74]	TACE + ICIs + Molecular Targeted Therapy (MTT) + 125I Irradiation Stent Placement in HCC with Main (Vp4) PVTT	Vp4 (main trunk)	TACE + immune checkpoint inhibitor(s) + targeted therapy + stent with ^125I (irradiated) after stent placement to maintain portal flow	Multicenter cohort/possibly comparative (with/without stent + TACE + ICI + MTT)	Recruiting; target 444	Primary: safety efficacy; secondary: OS, PFS, ORR, disease control; portal vein patency, etc.
NCT05166239 [75]	HAIC + Lenvatinib + PD-1 Inhibitors vs. Lenvatinib + PD-1 in Advanced HCC with PVTT	PVTT (extent not fully specified in public summary)	Hepatic arterial infusion chemotherapy (HAIC) + lenvatinib + PD-1 inhibitor vs. lenvatinib + PD-1 inhibitor alone	Phase 2, controlled (two arms)	Recruiting; total 66 patients (33 per arm)	Primary: 6 months PFS rate; secondary: OS, ORR, PFS, TTP, safety
NCT07062055 (BRAVE Study) [76]	QL1706 + Bevacizumab + SBRT for BCLC-C HCC with PVTT or Oligometastases	PVTT (includes PVTT; Vp classification not clearly specified)	ICI (QL1706) + anti-VEGF (bevacizumab) + SBRT (radiotherapy)	Phase 2, single arm, prospective multicenter	Planned enrollment: 46 patients; recruiting; start July 2025; estimated completion 2029	Primary: PFS; secondary: OS, ORR, DCR, duration of response, QoL

## Data Availability

No new data were created or analyzed in this study.

## Supplementary Materials

The following supporting information can be downloaded at https://www.mdpi.com/article/10.3390/cancers18040627/s1: Figure S1: PRISMA 2020 flow diagram for new systematic reviews which included searches of databases and registers only. Supplementary S2: The list of authors in group.
