# Supplementary material for: Immunotherapy in Hepatocellular Carcinoma with Portal Vein Tumour Thrombosis: From Poor Prognosis to Curative-Intent Strategies
Source: Cancers (Basel). 2026 Feb 14;18(4):627. doi: 10.3390/cancers18040627 (PMC12939993; doi:10.3390/cancers18040627)
Supplement: Supplementary file 1 [file cancers-18-00627-s001.zip › cancers-4117586-supplementary.pdf]

PRISMA 2020 flow diagram for new systematic reviews which included searches of databases and registers only

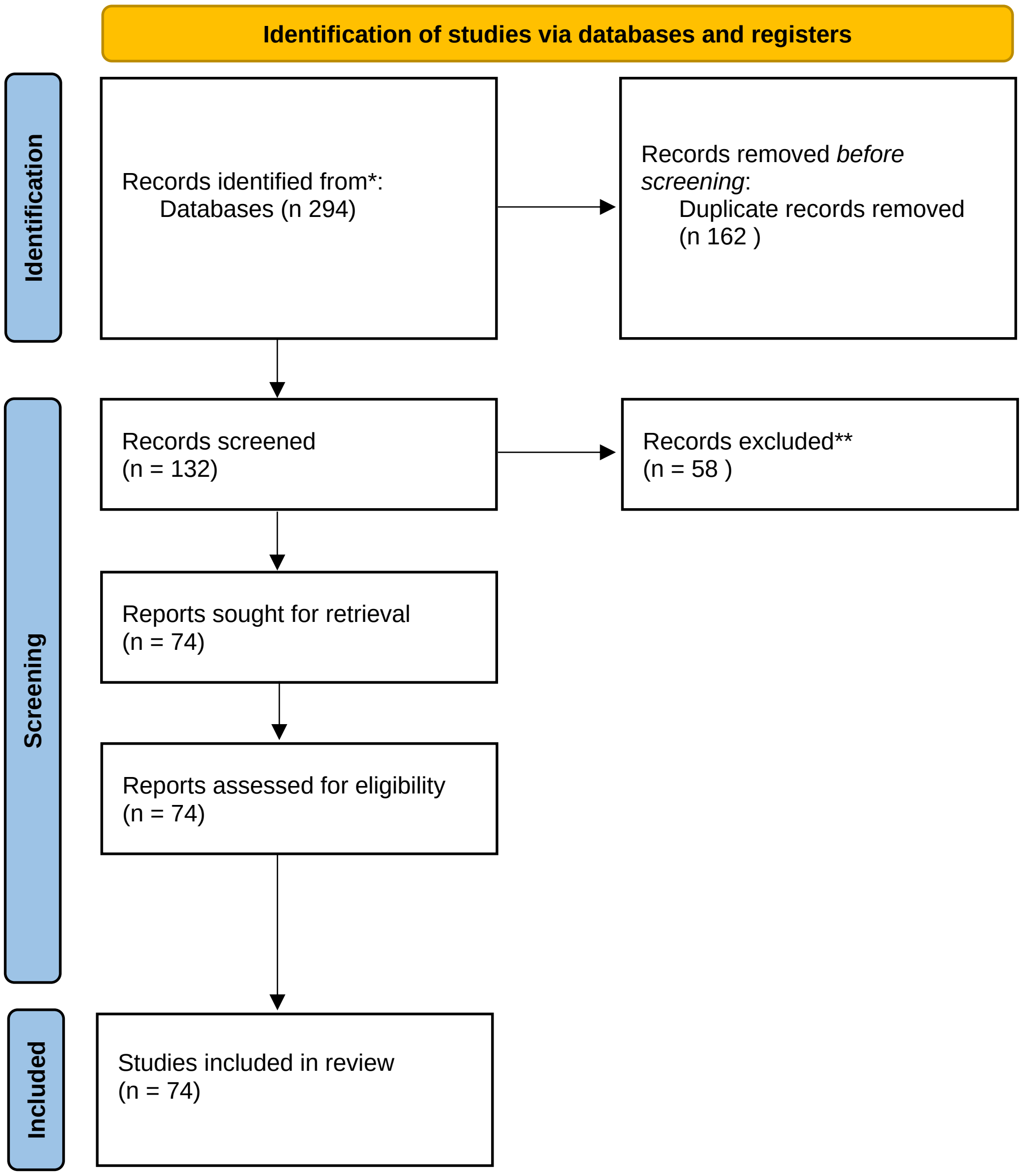

*Consider, if feasible to do so, reporting the number of records identified from each database or register searched (rather than the total number across all databases/registers).

**If automation tools were used, indicate how many records were excluded by a human and how many were excluded by automation tools.

Source: Page MJ, et al. BMJ 2021;372:n71. doi: 10.1136/bmj.n71.

This work is licensed under CC BY 4.0. To view a copy of this license, visit https://creativecommons.org/licenses/by/4.0/

PRISMA 2020 flow diagram for new systematic reviews which included searches of databases and registers only
Supplementary S2 The list of authors in group

List of authors/collaborators of the A.I.G.O. (Italian Association of Hospital Gastroenterologists)

Luca Marzi MD, Department of Gastroenterology, Bolzano Regional Hospital (SABES-ASDAA), 39100 Bolzano-Bozen, Italy; luca.marzi@sabes.it

Andrea Mega MD, Department of Gastroenterology, Bolzano Regional Hospital (SABES-ASDAA), 39100 Bolzano-Bozen, Italy; andrea.mega@sabes.it

Chiara Turri MD, Department of Gastroenterology, Bolzano Regional Hospital (SABES-ASDAA), 39100 Bolzano-Bozen, Italy; chiara.turri@sabes.it

Monica Zoeschg MD, Department of Gastroenterology, Bolzano Regional Hospital (SABES-ASDAA), 39100 Bolzano-Bozen, Italy; monica.zoeschg@sabes.it (M.Z.)

Rodolfo Sacco MD, Gastroenterology and Digestive Endoscopy Unit, Foggia University Hospital, Foggia, Puglia, Italy; rsacco@ospedaliriunitifoggia.it

Luisa Siciliani MD, Division of Clinical Immunology and Infectious Diseases, Fondazione IRCCS Policlinico San Matteo, Pavia, Italy; l.siciliani@smatteo.pv.it

Saveria Lory Crocè MD, Department of Medical, Surgical, and Health Sciences, University of Trieste, Trieste, Italy; lcroce@units.it

List of authors/collaborators of the C.L.E.O. (Italian Association of Hospital Hepatologists)

Luca Marzi MD, Department of Gastroenterology, Bolzano Regional Hospital (SABES-ASDAA), 39100 Bolzano-Bozen, Italy; luca.marzi@sabes.it

Andrea Mega MD, Department of Gastroenterology, Bolzano Regional Hospital (SABES-ASDAA), 39100 Bolzano-Bozen, Italy; andrea.mega@sabes.it

Rodolfo Sacco MD, Gastroenterology and Digestive Endoscopy Unit, Foggia University Hospital, Foggia, Puglia, Italy; rsacco@ospedaliriunitifoggia.it

Luisa Siciliani MD, Division of Clinical Immunology and Infectious Diseases, Fondazione IRCCS Policlinico San Matteo, Pavia, Italy; l.siciliani@smatteo.pv.it
